# Applications of artificial intelligence and deep learning in molecular imaging and radiotherapy

**DOI:** 10.1186/s41824-020-00086-8

**Published:** 2020-09-23

**Authors:** Hossein Arabi, Habib Zaidi

**Affiliations:** 1 Division of Nuclear Medicine and Molecular Imaging, Geneva University Hospital, CH-1211 Geneva 4, Switzerland; 2 Geneva University Neurocenter, Geneva University, CH-1205 Geneva, Switzerland; 3 Department of Nuclear Medicine and Molecular Imaging, University of Groningen, University Medical Center Groningen, 9700 Groningen, RB Netherlands; 4 Department of Nuclear Medicine, University of Southern Denmark, 500 Odense, Denmark

**Keywords:** Molecular imaging, Radiation therapy, Artificial intelligence, Deep learning, Quantitative imaging

## Abstract

This brief review summarizes the major applications of artificial intelligence (AI), in particular deep learning approaches, in molecular imaging and radiation therapy research. To this end, the applications of artificial intelligence in five generic fields of molecular imaging and radiation therapy, including PET instrumentation design, PET image reconstruction quantification and segmentation, image denoising (low-dose imaging), radiation dosimetry and computer-aided diagnosis, and outcome prediction are discussed. This review sets out to cover briefly the fundamental concepts of AI and deep learning followed by a presentation of seminal achievements and the challenges facing their adoption in clinical setting.

## Introduction

Artificial intelligence (AI) has attracted considerable attention during the last few years, although it has been around since a few decades. With the introduction of deep learning algorithms, research focusing on multimodality medical imaging has increased exponentially; targeting mainly applications deemed to rely on human intervention/interpretation or handcrafted data preparation/modification (Sim et al. 2020). These algorithms exhibited tremendous potential to effectively learn from data, correctly interpret the data, and successfully accomplish certain tasks following appropriate training. AI is gaining momentum in medicine in general, owing to effective handling of the data overflow, eliminating optimism bias coming from false human generalization based on the individual experiences, management of rare diseases (or frequently overlooked cases), robustness to inter- and intra-person/center variations, and the possibility of being perfectly up-to-date with minor modifications (Nensa et al. 2019).

This paper sets out to discuss the conceptual basis of artificial intelligence and its potential clinical applications. The main focus is on the major applications of AI, in particular deep learning approaches, in molecular imaging and radiation therapy fields. In this regard, five generic areas where AI-based solutions have attracted attention and are considered as game-changer or paradigm shifter were identified. The primary aim of this work is to give a general insight into the current status of AI technology in molecular imaging and radiation therapy through reviewing seminal works and novel frameworks proposed in each of the generic fields. Moreover, the challenges and barriers faced by developers/scientists on the way of full-scale implementation of AI-based solutions in the clinic and promising research avenues that require additional research and development efforts are discussed.

As discussed earlier, AI was proposed to undertake certain tasks in any of the four phases of nuclear medicine examinations. Overall, AI has the potential to effectively contribute in specific areas in molecular imaging owing to its promising/superior performance or the desire to upgrade/enhance current techniques for more accurate examinations. The applications of artificial intelligence in molecular imaging and radiation therapy are summarized in five successive sections followed by the last section (the “Challenges and opportunities” section) where the challenges and opportunities of AI systems in these fields are discussed. A brief overview of machine learning and deep learning techniques as applied to nuclear medicine is provided in the “Principles of machine learning and deep learning” section. The “PET instrumentation” section presents recent AI-based developments in PET instrumentation with focus on timing and event localization of the PET detectors. The “PET image reconstruction/quantification/segmentation” section talks about the state-of-the-art AI algorithms developed for the tasks of PET image reconstruction, quantification, and segmentation. Some applications and algorithms covered in this section are also applicable to radiation therapy such MR-based synthetic CT generation and organ segmentation. The “PET image denoising” section is dedicated to PET image denoising and algorithms enabling to predict high quality/standard-dose images from low-dose scans in both PET and SPECT imaging. The “Radiation dosimetry calculation” section discusses the applications of AI techniques in radiation dosimetry, which is valuable in both diagnostic molecular imaging as well as molecular radiotherapy. The “Computer-aided diagnosis and outcome prediction” section focuses on more generic topics of computer-aided diagnosis and outcome prediction related to the characterization of malignant lesions using AI techniques.

## Principles of machine learning and deep learning

Deep learning algorithms are categorized into two main classes: supervised and unsupervised techniques. In supervised learning, the ground truth or desired outputs associated with the inputs are available within the training, wherein a specific end-to-end transformation and/or association is established to predict the desired outputs for new inputs. Special attention should be devoted to avoid overfitting, which is an ineffective learning process mostly relying on the memorization of the example data. A number of studies have reported relatively small errors (highly accurate results) when applying deep learning approaches on a specific dataset which might be due to the consequence of the overfitting issue as the external test/validation dataset highly resembles the training dataset. Under more challenging conditions (test dataset with large variability), such a good outcome is less likely to be observed (Sahiner et al. 2019).

**Fig. 1:**
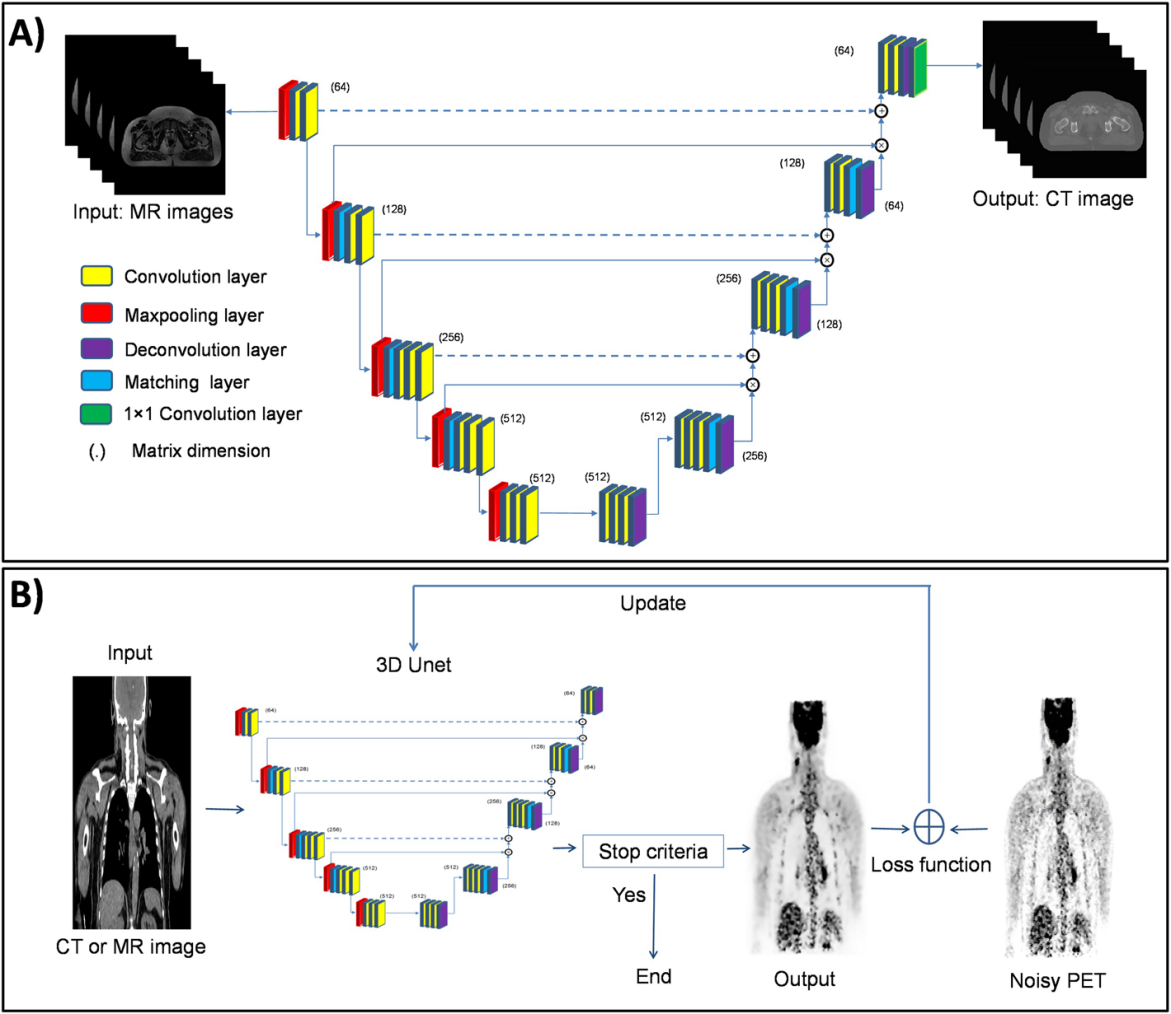
Examples of (**a**) supervised and (**b**) unsupervised deep learning approaches employed in molecular imaging for the task of MRI-based synthetic CT generation (**a**) and PET denoising (**b**). In supervised learning, the model is trained using a labeled dataset, providing answer keys based on which the accuracy of the model can be evaluated within the training process. In contrast, in unsupervised learning, the algorithm tends to make sense of unlabeled data relying on the extraction of dominant features and patterns on its own

Supervised convolutional neural networks (CNNs) commonly consist of an input layer which simply accepts the input data in its original dimension, feature extraction layers with repeating pattern of convolutional operators to extract the underlying features of the input data and progressively create higher-order discriminative features and classification or output layer, usually consisting of multiple layers to synthesize the output data or produce class probabilities based on the high-order features. This architecture is referred to as U-net owing to the successive convolutional layers/operators which create a U shape (Fig. 1a). Various CNN architectures for supervised training schemes are available, including VGGNet (Simonyan and Zisserman 2014), GoogLeNet (Szegedy et al. 2015), and ResNet (He et al. 2016). Recurrent and/or recursive neural networks (RNNs) are among the supervised deep learning algorithms, which in contrast to CNN models, have the ability to access/send/process information over time steps. RNNs take sequences of input vectors to model them one at a time enabling both parallel and sequential processing appropriate for data analysis in time series, such as dynamic or longitudinal studies (Wang et al. 2019a).

Conversely, in unsupervised learning, the machine learns from the input dataset itself without any labels through decoding the inherent distinctive structures/patterns within the input data. In many clinical applications where the cost of generating paired dataset (input/label) is prohibitively high, unsupervised training could offer acceptable solutions. However, the majority of research reported in the literature exploited supervised training owing to relatively easier training (but no data preparation) and straightforward evaluation owing to the availability of the ground-truth labels (Sahiner et al. 2019). In addition, a combination of these two approaches (supervised and unsupervised) was suggested when obtaining a sufficient number of labeled data for supervised training is prohibitively difficult. In many cases, unlabeled data/images are readily available; however, the creation of annotated data/images requires considerable investment in time and experts knowledge. Building a model based on a small number of annotated data/images would not normally lead to satisfactory outcomes. Hence, semi-supervised techniques make use of large unlabeled dataset to learn the underlying structure of the data which will be completed (fine tuned) using the labeled dataset and task-specific training (Chen et al. 2019).

The major architectures for unsupervised deep learning include autoencoders and generative adversarial networks (GANs). An autoencoder consists of three major components: an encoder, code or embedding, and a decoder wherein the encoder part compresses the input data into a number of fixed (in terms of dimension) codes or vectors and then the decoder component transforms the fixed codes into the same input data. Autoencoders are often employed as a sub-network of a larger model, which is also able to serve as a standalone network. GANs are considered as an adept at synthesizing novel/new data relying on different training datasets (Creswell et al. 2018). GANs consist of discriminator and generative networks wherein the generator (generative network) is trained to synthesize plausible data. The discriminator, which is a typical CNN, attempts to distinguish fake data generated by the generative network from real data. Variations of GANs, such as cycleGAN, are very powerful tools in unsupervised image translation, such as MRI to CT conversion (Armanious et al. 2019).

Overall, the applications of AI in medicine could be associated with two different realms of activities. The first is task delegation, wherein physicians’ everyday tasks consisting of routine operations, verifications, and data preparation could be delegated to AI, offering the human resources more time to achieve higher-value tasks (Hainc et al. 2017). The day-to-day tasks of medical imaging involve a large number of activities, in particular manual handling of the data and information, which tends to be error prone and requires too little creativity and intellectual efforts from the experienced workforce. AI could be an appropriate substitute for the human workforce with the advantage of zero likelihood of forgetting or non-properly performing a task. Second is the black-box specialist, wherein AI is considered as a superhuman capable of performing special tasks with precision and accuracy beyond the human capacity (Liu et al. 2019a).

One could categorize a typical nuclear medical imaging pipeline into four different phases, including plan definition, image acquisition, outcome interpretation, and diagnosis/reporting. AI could effectively facilitate and/or enhance the quality of the workflow in each of these phases (Nensa et al. 2019). An examination of the patient starts with the definition of a medically defined and indicated procedure. The more invasive, hazardous, and/or expensive the procedure is the more care and stricter guidelines are required. AI could potentially reduce the burden of examination planning in a cost-effective manner (Ansart et al. 2020), and more interestingly eliminate the ever-present risk of human errors. For instance, based on patient’s history, pathological and/or demographical descriptors, previous examinations and present symptoms, a series of clinical investigations can be proposed by an AI expert system to aid the clinicians/physicists in their examination planning (such as prescription of specific clinical tests or imaging exams). Image acquisition/processing in nuclear medicine is witnessing notable technical improvements taking advantage of modern imaging technology and recent advances in machine learning. The feasibility of inter-and intra-modality image translation could potentially open new avenues in image acquisition procedures and potential clinical applications (Han 2017; Arabi et al. 2019; Lee et al. 2020; Kaji and Kida 2019). Moreover, improvements in PET image quality in terms of spatial resolution and noise properties have been active research topics aiming at achieving shorter acquisition times (lower injection dose) (Xiang et al. 2017; Wang et al. 2019b) and higher temporal resolution in dynamic PET imaging (Cui et al. 2017). Clinical data collection commonly involves a list of examinations that should be chronologically performed. However, in some cases, prompt actions should be prioritized according to certain findings. In such a scenario, AI could be employed to raise alerts and/or modify/extend the examinations to take into account the unexpected findings (Prevedello et al. 2017). The capability of automated diagnosis or prediction of rare/unknown outcomes has been closely linked to the superhuman performance of AI. Micro-metastases (early metastatic disease) detection, prediction of survival or response to therapy, and in general identification of complex cases and/or rare diseases are among the major applications of AI in nuclear medicine (Ellmann et al. 2019; Hustinx 2019). Figure 1 illustrates two examples of the utilization of deep learning methods in molecular imaging in supervised and unsupervised learning. Figure 1a depicts the structure of a typical U-Net network used for synthetic CT generation from MR sequences (similar to approaches used in (Han 2017; Arabi et al. 2018)). The synthetic CT images are generated from the corresponding MR sequences in an end-to-end mode owing to the presence of the ground truth within the training phase. A novel unsupervised PET denoising technique is depicted in Fig. 1b wherein prior information in MR or CT images is used to distinguish the underlying signals in PET images from the noise distribution in an unsupervised mode (no ground truth exists for the denoised PET images) (Cui et al. 2019).

## PET instrumentation

The first and foremost important issue in a PET scanner is to detect the high-energy annihilation photons (511 keV) with a high sensitivity. Besides, accurate measurement of photons energy (deposited in the PET detectors), the arrival time of the coincidence photons, and the location where the interaction between the photons and detection medium occurred, play critical roles in the performance of the PET system. These parameters largely influence the sensitivity, spatial resolution, time-of-flight (TOF) capability, scatter and random corrections. In this regard, estimation of the photon interaction location within the detector modules (position of interaction) and extraction of the timing information of the coincidence photons (time of interaction) have been the most prevalent targets of AI-based solutions in PET instrumentation (Gong et al. 2019a).

Conventional PET scanners have been designed using scintillation crystals coupled to photomultiplier tubes (PMTs), thus inherently limiting the performance of PET scanners. The dependency on high voltage supply besides their bulky size and high sensitivity to temperature variation, humidity, and magnetic fields are the main disadvantages of PMTs. Conversely, silicon PMTs (solid-state semiconductors) as main components of digital PET scanners (Schillaci and Urbano 2019), offer significant technical advantages, such as one-to-one crystal and detector coupling, higher spatial resolution, enhanced TOF capability, and shorter dead-time. Digital semiconductor photodetectors are ideal for building hybrid PET/MRI scanners owing to their low sensitivity to magnetic fields (Zaidi and Becker 2016). Though solid-state semiconductor detectors resolved some of the limitations of conventional PET detectors, AI algorithms can be employed in these technologies to offer higher spatial and energy resolution, noise reduction, and improve timing performance (Zatcepin et al. 2020).

### Event positioning

**Fig. 2:**
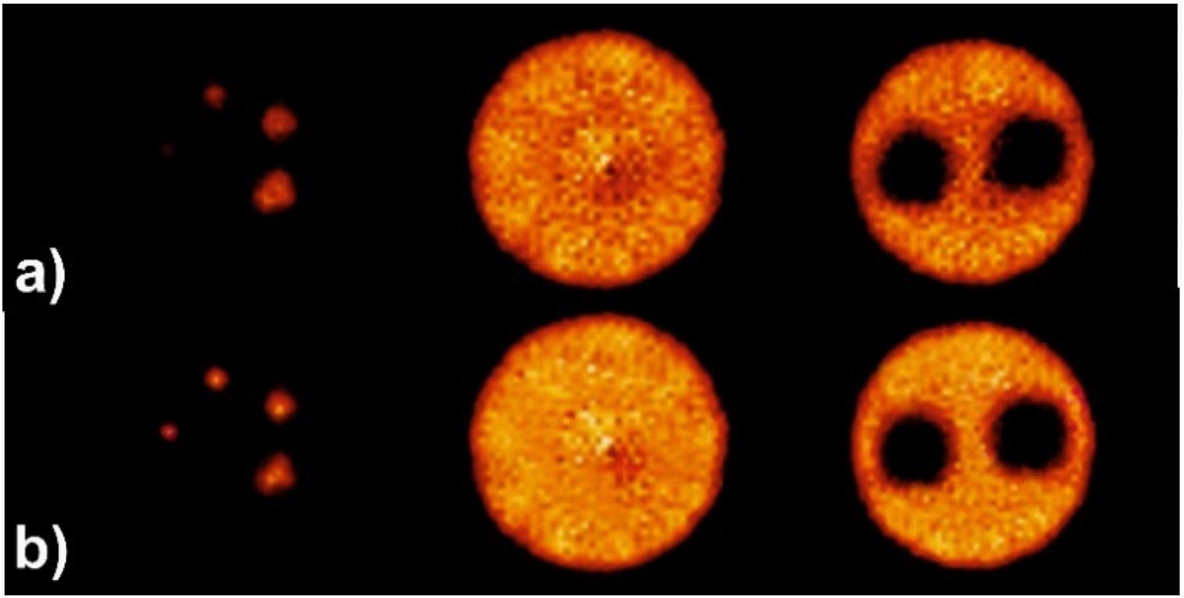
Representative slices through the image quality phantom produced for a PET scanner using (**a**) Anger positioning logic and (**b**) deep learning-based algorithm. Reprinted with permission from MDPI (Sanaat and Zaidi 2020)

Almost in all PET detectors, apart from systems equipped with semiconductors, the location where the photon interacts in the detector is estimated through processing of the scintillation light distribution collected by the photodetectors (attached to the PET detectors). However, singularities, such as nonlinear light distribution close to the edges, multiple reflections of the scintillation light within the detector medium, multiple Compton interactions of the annihilation photons in the PET detector, statistical uncertainty of light detection and noise have rendered the task of positioning a complicated problem for analytical models. In this regard, the primary aim of AI approaches is to provide superior localization of the position of interaction compared to conventional methods, such as center-of-gravity (COG) estimation, in the presence of noise and limited statistics of light distribution within the PET detectors. Positioning algorithms for estimation of the photon interactions are required for both monolithic and pixelated detectors, wherein commonly supervised machine learning approaches are trained to achieve this task. This requires labeled training dataset, which are normally obtained through irradiation of the PET detectors with a point and/or pencil-beam of 511-keV photons at different angles or positions with a known beam geometry. Then, AI techniques can be employed to decode and/or extract the exact position of photon interactions within the PET detectors from the light distribution over the photodetectors, such as silicon photomultipliers (SiPMs). Moreover, implementation of deep learning-based positioning would not add computational burden compared to other algorithms, such as maximum likelihood estimation methods, and benefits from lower complexity for execution on graphical processing units (GPUs). In this regard, a convolutional neural network (CNN) was trained to map the light distribution over SiPMs (charge collected from each channel of the SiPMs) as input to 2D position-of-interaction as classification output for a quasi-monolithic detector (Peng et al. 2018). Regarding other position estimators, such as COG, machine learning techniques resulted in superior spatial resolution owing to the reduced positioning bias, particularly at the edges of the PET detectors (Müller et al. 2018). Likewise, Sanaat and Zaidi proposed a deep neural network for estimation of the depth of interaction in monolithic scintillation crystals coupled to SiPMs. The proposed approach improved the spatial resolution of the simulated monolithic scintillation detector by up to 26% (from 1.38 mm to 1.02 mm) compared to Anger logic positioning (Sanaat and Zaidi 2020). Figure 2 illustrates representative slices of the image quality phantom where Anger positioning logic and the proposed deep learning-based algorithm were employed for event localization. Improved spatial resolution and signal to noise ratio are seen in both hot and cold spots.

In contrast to monolithic PET detectors, few investigations of AI approaches have been carried out on pixelated detectors since analytical techniques, such as the Anger logic (together with a lookup table) are able to precisely estimate the position of interaction. Nevertheless, the challenges of inter-crystal scatter, reduced energy resolution, estimation of the depth of interaction, and low light output are still faced by pixelated detectors, wherein AI could offer promising solutions. In this regard, the LabPET™ scanner exploited a neural network method to detect inter-crystal scatter (Michaud et al. 2014). This approach, in the first step, identifies the triple coincidences that most likely steam from inter-crystal interactions through analysis of the deposited energy and interaction positions. Monte Carlo simulations were used for the training of the network to estimate the most likely lines-of-response (LORs) for the scattered photons within the PET detector. This approach led to 54% increase in sensitivity of the scanner.

The limitations faced by AI algorithms for the localization problem lies in the generation of training datasets. The uncertainty associated with the location of the first and/or actual interaction within the PET detector, high probability of multiple Compton scattering prior to photoelectric absorption and discrepancy between ground truth (training dataset) generated by Monte Carlo simulations and true experimental measurements are the major challenges of AI-based solutions for position-of-interaction estimation.

### Time-of-flight measurement

#### Timing

In addition to the position of interaction, the time of interaction is the most important measure provided by photodetector signals, which significantly impacts the TOF capability and the accuracy of random coincidence rejection. Estimation of the time of interaction is highly challenged by the low light output of most PET detectors and the fact that only the first few scintillation photons determine the true interaction time. Currently, in most PET scanners, the time of interaction is determined through a simple measure of the time at which the signal generated by the photodetector crosses a certain threshold. This method overlooks many features prevailing in photodetector signals, such as the shape or rising edge which provide information about the true time of coincidence in the detector. This is a prominent motivation for the application of AI in PET signal processing.

In this context, Berg et al. estimated TOF information directly from two lutetium yttrium oxyorthosilicate (LYSO) detectors (coupled to PMTs) using a CNN with minimal human intervention (Berg and Cherry 2018). To this end, ground-truth (training) data were created through scanning a ^68^Ga point source stepping with increments of 5 mm between the PET detectors. Significant improvement in TOF resolution was observed using this technique compared to two conventional methods, namely, leading edge and digital constant fraction discriminator (CFD). The CNN approach led to a TOF resolution of 185 ps vs 231 and 242 ps achieved by leading edge and CFD methods, respectively.

AI algorithms have to be implemented on fast front-end electronic devices, such as FPGA, to avoid expensive storage and offline processing of the raw data. In addition, re-calibration (in other words re-training) of AI approaches requires time-consuming acquisition of labeled (training) dataset through dedicated experimental designs. For instance, for TOF estimation, AI needs adequate training dataset set over a complete range of TOF differences (Berg and Cherry 2018).

The success of CNN approaches in accurate estimation of the position of interaction as well as time of interaction suggests an effective all-in-one AI estimator with overall superior performance in localization, timing, and energy discrimination in the future.

## PET image reconstruction/quantification/segmentation

### Image reconstruction

The ALARA (as low as reasonably achievable) principle inspired the minimization of the injected activity in PET imaging. Hence, PET images suffer from typical high noise characteristics owing to statistical uncertainties associated with the emission and detection of coincidence photons, as well as the ill-posed nature of the image reconstruction problem. AI approaches, in particular deep learning algorithms, have been exploited in the field of PET image reconstruction to address the abovementioned issues (Zhu et al. 2018). Advanced PET image reconstruction frameworks incorporate a penalized likelihood term or/and prior knowledge, governed by hyperparameters within image reconstruction to suppress the noise (Mehranian et al. 2017). AI algorithms could mainly replace the entire penalty term, which makes reconstruction robust to various noise levels through efficient modeling of the underlying process to render the intermediate reconstructed images well matched to the measured data (Gong et al. 2019b; Kuang et al. 2019). Deep learning-based image reconstruction, in comparison to kernel-based methods (Wang and Qi 2014), is able to take advantage of subject-specific priors (inter-subject prior information), thus enabling to model the underlying image quality degradation factors (Xie et al. 2019). In addition, the incorporation of deep learning methods within the reconstruction algorithm enabled to establish an end-to-end mapping of the PET data from sinogram space to the image domain, wherein iterative image reconstruction would be completely replaced by a fast and seemingly black-box deep learning network (Haggstrom et al. 2019). Since the physics of PET is overlooked (or at least not explicitly exploited), this approach faces the challenge of sufficiently large training data collection representing the underlying physical processes.

### Quantitative imaging

In addition to image reconstruction, corrections for physical degrading factors, such as attenuation, scatter, and partial volume effect challenge the quantitative potential of PET imaging. AI has recently proposed new approaches to cope with these issues in an efficient way, particularly in the absence of concurrent anatomical information to guide the process.

In an adult scan, only 10% of the annihilation photons could escape from the body without undergoing interaction with biological tissues. Therefore, taking photon attenuation into account plays a vital role to achieve quantitative PET imaging. In hybrid PET/CT scanners, the attenuation correction (AC) map is readily provided by CT images. However, in hybrid PET/MR or standalone PET scanners, there is no straightforward approach to obtain an AC map. Apart from segmentation-, atlas-based and joint emission and transmission reconstruction approaches, AI algorithms have been recently proposed to tackle the challenge of generating AC maps from single or multiple MR sequences (Han 2017; Arabi et al. 2019). Synthetic CT generation from MR sequences resembles the concept of image transfer used in computer vision applications. The only major distinction is that style transfer algorithms for natural images focus on general structures and signature properties; however, synthetic CT generation requires quantitative accuracy, thus local intensity prediction plays a key role. Overall, deep learning approaches seem to exhibit better (at least comparable) performance for PET quantification compared to existing state-of-the-art approaches in whole body (Arabi and Zaidi 2019; Hwang et al. 2019), pelvic (Arabi et al. 2018; Torrado-Carvajal et al. 2019), and brain imaging (Liu et al. 2017; Gong et al. 2018; Blanc-Durand et al. 2019). These approaches require at least one MR sequence as input for CT synthesis, whereas deep learning-based joint estimation of attenuation and emission images from TOF PET raw data (Hwang et al. 2019; Hwang et al. 2018) and direct scatter and attenuation correction in the image domain (Yang et al. 2019; Bortolin et al. 2019; Shiri et al. 2020a; Arabi et al. 2020), and sinogram domain (Arabi and Zaidi 2020) could possibly obviate the need for any structural/anatomical images. It should be noted that the information provided by CT images is not ideal for PET attenuation correction owing to the discrepancy between photon energies used in CT and PET imaging. The attenuation coefficients provided by polychromatic CT images (using for instance 120 kVp) should be converted to attenuation coefficients at a monochromatic energy of 511 keV. Moreover, the primary photon interaction in CT imaging is photoelectric effect, whereas Compton scattering dominates photon interactions in PET imaging owing to the relative higher photon energy. These issues challenge the accurate conversion/scaling of attenuation coefficients obtained from CT for PET attenuation correction.

**Fig. 3:**
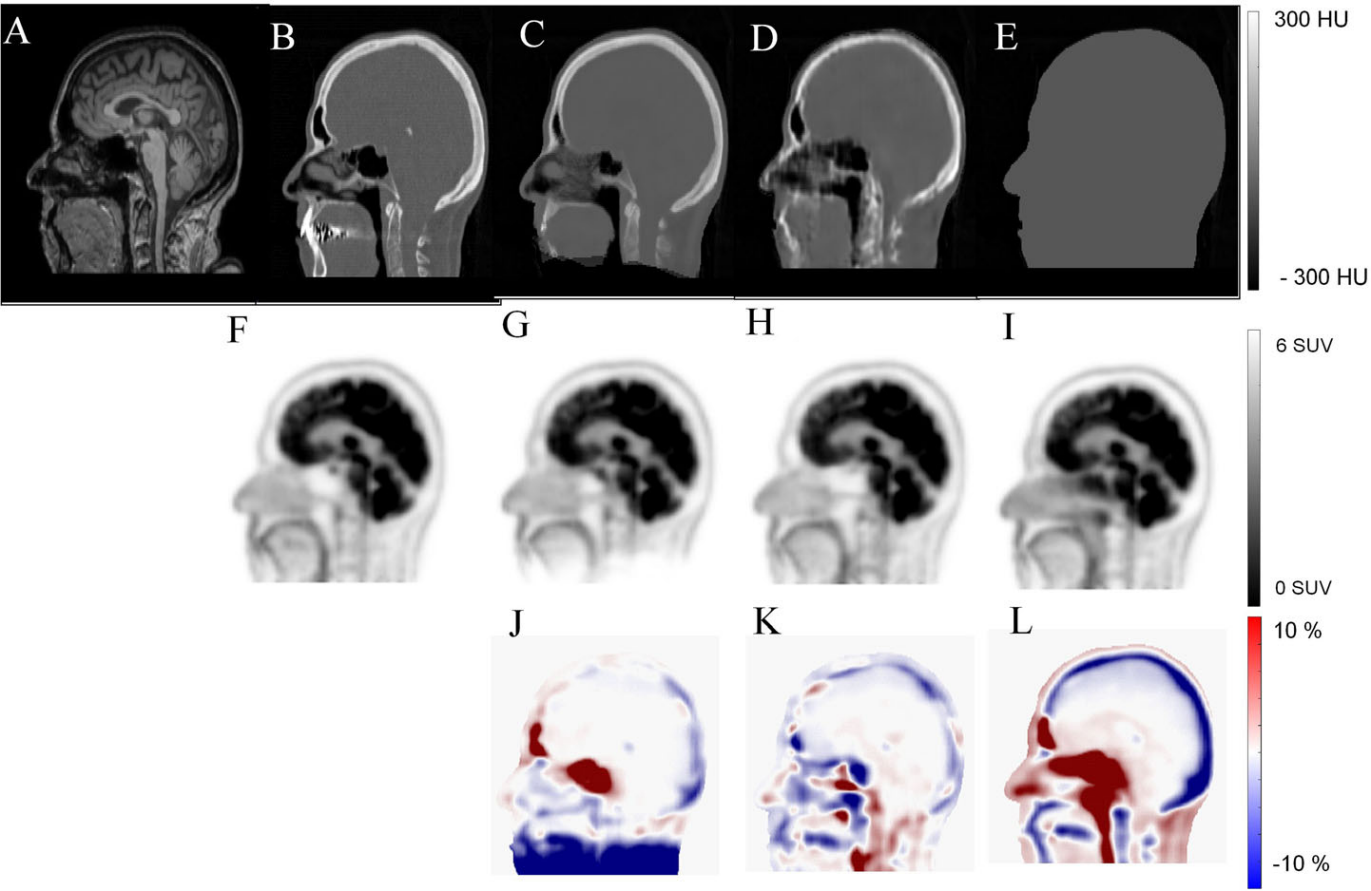
Synthetic CT generation from T1-weighted MR image using a generative adversarial network. **a** Target MRI, **b** reference CT, **c** atlas-based, **d** deep learning-based, and **e** segmentation-based synthetic CT images. PET ages corrected for attenuation using **f** reference CT, **g** atlas based, **h** deep learning based, and **i** segmentation-based synthetic CT images. PET bias map (relative error in %) for **j** atlas based, **k** deep-learning based, and **l** segmentation-based attenuation correction

Figure 3 compares synthetic CT images estimated from T1-weighted images using a generative adversarial network (Arabi et al. 2019), atlas based (Arabi et al. 2016), and segmentation-based methods. PET images corrected for attenuation and scatter using the abovementioned synthetic CT images along with the corresponding bias maps are provided for quantitative assessment.

Estimation of photons scattered within the patient challenges quantitative PET reconstruction owing to the stochastic nature of photon interaction with biological tissues with its modeling requiring both knowledge of the activity distribution and attenuation map. Monte Carlo simulation is considered the gold standard tool for scatter estimation; however, this approach is prohibitively time-consuming to generate results with reliable statistics. Though the single-scatter simulation (SSS) algorithm (widely used on software implemented on commercial PET scanners) is remarkably less time consuming, it only assumes the detection of single-scatter events. Hence, there is ample scope for improvement (considering multiple scattering and TOF information). In this regard, ongoing research aims at applying AI for fast and accurate scatter estimation. Scatter modeling in PET is more challenging in the pelvis region due to the high activity concentration in the bladder and FOV limitations (the coverage of the FOV might not be sufficient to include all significant out-of-body scattered events) (Berker et al. 2018). The advantages of AI for scatter estimation is the fast computational time and likely higher quantitative accuracy (compared to model-based scatter estimation) provided robust training using ground truth data from accurate Monte Carlo simulations could be generated in a reasonable time.

Attenuation and scatter correction enable quantitative analysis and improve the quality of SPECT images. Standalone SPECT cameras face the challenges of quantification and susceptibility to attenuation artifacts. In this regard, Shi et al. proposed a novel deep learning-based framework for estimation of the attenuation maps from SPECT data (Shi et al. 2020). This framework relies on both photopeak and scatter windows of SPECT images (based on the recorded energy of the photons) to extract the latent attenuation information from SPECT emission data. Moreover, in SPECT/CT imaging, a major challenge facing quantitative SPECT imaging for some radionuclides, such as ^90^Y, is the lack of accurate scatter estimation since a simple energy-based windowing (widely used in clinical practice) would not lead to satisfactory outcome. Xiang et al. proposed a deep learning-based scatter estimator for SPECT/CT imaging to replace computationally expensive Monte Carlo (Liu et al. 2017) simulations (Xiang et al. 2020). The deep learning model was trained to estimate scatter projections from ^90^Y SPECT images in the projection domain and accompanying CT images. The proposed technique exhibited good agreement with MC simulation results with less than 40 s computation time compared to 80 min required by lengthy MC simulations.

### Image segmentation

AI algorithms have been largely employed for organ delineation as well as lesion segmentation using different imaging modalities. To this end, two different frameworks are commonly used, including taking the whole image as input or confining the input to only a subset of the image containing the target organ/lesion (Sahiner et al. 2019; Liu et al. 2019b). Organ/structure segmentation is used to calculate clinical relevant parameters or to confine the search space for the task of computer-aided detection or radiation treatment planning (Hesamian et al. 2019). Lesion segmentation is more challenging than organ segmentation, though they are technically very similar, due to the large variability in shape and size of malignant lesions. Prior to radiotherapy planning, the target treatment volume should be accurately delineated. This process tends to be particularly difficult (tedious) and time consuming and prone to large inter- and intra-observer variability if performed manually. Automatic nasopharyngeal carcinoma tumor segmentation from ^18^F-FDG PET/CT scans using a U-Net architecture proved the feasibility of this task (dice coefficient = 0.87) using AI-based algorithms (Zhao et al. 2019a). Similar studies on head and neck (Huang et al. 2018), as well as lung cancers (Zhao et al. 2018) exhibited promising results using convolutional neural networks for automated tumor segmentation from PET/CT images. Nevertheless, fully automated lesion delineation from PET, CT, and MR images or any combination of these images still remains a major challenge owing to the large variability of lesion shape and uptake associated with various malignant diseases. This necessitates the collection of adequate large amounts of training dataset to properly cover the population distribution, wherein labor-intensive manual segmentation might be inevitable (Cheplygina et al. 2019). Moreover, vendor-specific scanner performance, differences in PET image acquisition and reconstruction protocols, and difficulties associated with standardization further adds to the complexity of this problem.

## PET image denoising (low-dose scanning)

The presence of high noise levels in PET images adversely impacts lesion detectability and quantitative accuracy (by introducing noise-induced bias) leading to uncertainties in clinical diagnosis and staging of disease. Moreover, there are strong incentives to reduce injected activities of positron-emitting tracers in longitudinal and pediatric PET imaging studies, which further increases noise magnitude (Schaefferkoetter et al. 2019). Conventional post-reconstruction denoising methods tend to degrade the spatial resolution, which might hamper lesion detectability and identification of radiotracer uptake patterns.

Conversion of low-quality (low-dose) to high-quality (standard-dose) PET images can be considered as a regression problem. In this regard, AI algorithms can potentially offer promising solutions since the training dataset could be easily collected from clinical studies (PET listmode data) without the need for manually defined ground truth (Zhang et al. 2017). AI-based solutions, in particular deep learning approaches, have demonstrated superior performance compared to traditional denoising techniques for various radiotracers and clinical indications (Wang et al. 2018; Ouyang et al. 2019).

**Fig. 4:**
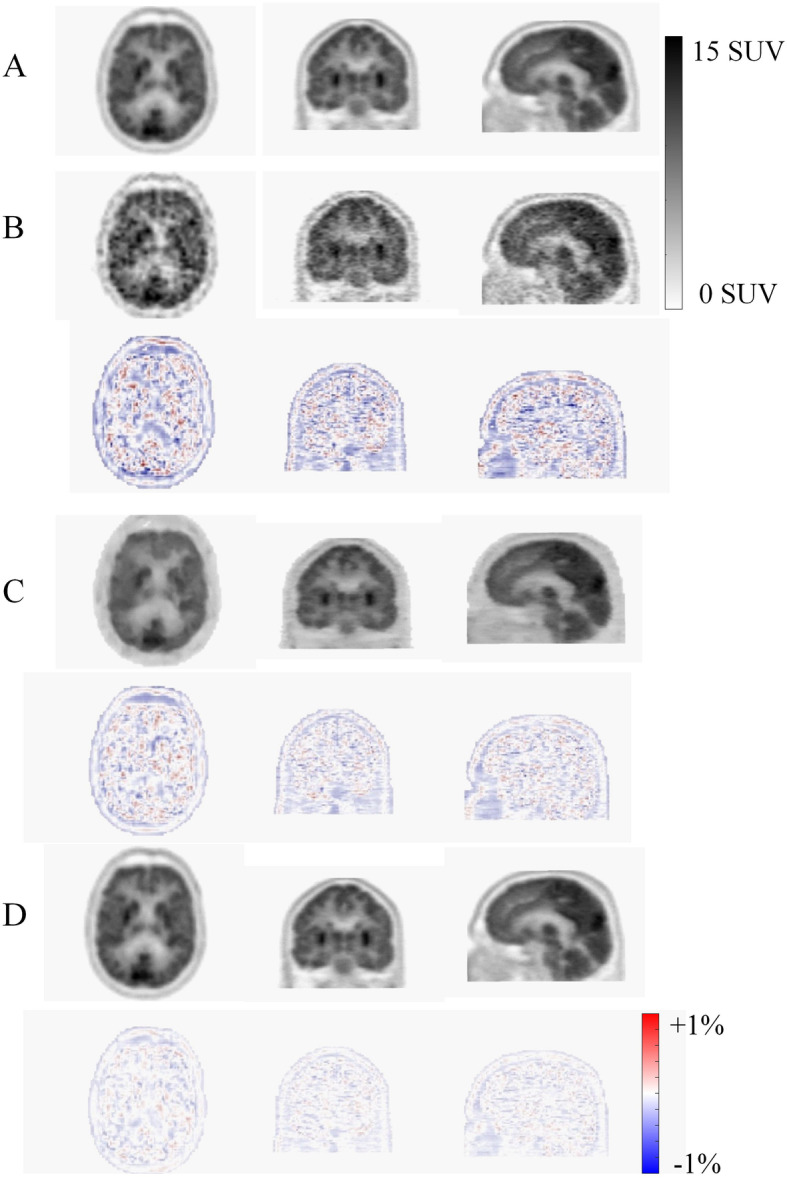
Standard PET (high dose) estimation from 5% low-dose PET image in the sinogram and image domain. **a** Reference standard PET image, **b** 5% low-dose PET image together with the corresponding PET bias map (bottom row), **c** Deep learning-based standard dose prediction in the image space and **d** in the sinogram space along with their corresponding bias maps, respectively

For deep learning approaches, the primary interest is in high-quality (standard-dose) PET image estimation from the corresponding low-quality (low-dose) image, thus enabling shorter PET scanning and/or reduced injected dose (Xu et al. 2017). In this regard, anatomical priors (mostly from MR images) can be incorporated into the denoising process to improve PET image quality. The advantage of deep learning approaches is that the additional channels/inputs can be easily introduced into the network without user-defined weight assignment or intervention. Multispectral MRI (providing multi-contrast images of the underlying biological tissues) has been shown to markedly enhance the quality of the outcome when incorporated into deep learning-based denoising approaches. For instance, Chen et al. demonstrated a full-dose estimation of amyloid brain PET images from a 1% low-dose acquisition. Incorporation of multi-contrast MR images, namely T1- and T2-weighted and T2-FLAIR, into the regression process improved the root mean square error (RMSE) of the estimated standard uptake value ratio (SUVR) from 0.20 to 0.15 (Chen et al. 2018). Along with noise suppression in the image domain, deep learning approaches have been applied in sinogram space to render high quality (standard dose) (Sanaat et al. 2020) as well as super-resolution PET images (Hong et al. 2018). Figure 4 depicts an example of standard PET (high dose) estimation from low-dose PET images (corresponding to 5% of the standard dose) derived following training in image and sinogram domains. Synthetic PET estimated in the sinogram space resulted in superior noise suppression and lower quantitative bias.

In addition to static PET imaging, the acquired PET data can be divided into predefined number of time frames to enable dynamic PET imaging, which in turn enables parametric imaging derived through application of a particular kinetic model. The quality of parametric images is much lower than static PET images owing to the fact that parametric images generated at the voxel level are noisier and that compartmental modeling is intrinsically an ill-posed problem. In this regard, AI algorithms can be used to process (denoise) dynamic PET frames prior to fitting to a kinetic model while taking advantage of the existing correlated information in neighboring time frames (Cui et al. 2017). In a more generalized approach, a deep neural network can be trained to translate dynamic PET frames into corresponding parametric images. This approach would be of special interest if one or more time frames from the PET data are missing or only a partial scan of the entire dynamic data is available. Scott et al. used a 30 min dynamic PET scan simultaneous to the cerebral blood flow extracted from accompanying MR acquisitions to estimate binding potential information, using a deep convolutional neural network, corresponding to a full 60-min PET scan (Scott et al. 2018).

Briefly, a number of studies employed deep learning algorithms for noise reduction in static and dynamic PET imaging demonstrating promising performance compared to state-of-the-art approaches. So far, most of the studies were conducted using conventional tracers, such as ^18^F-FDG. It appears that neural networks developed/trained using specific tracers and/or acquisition protocols are applicable to other radiotracers and protocols but further research is required to support this hypothesis (Liu et al. 2019c).

In SPECT myocardial perfusion imaging (MPI), two scenarios enabling to reduce the acquisition time and/or injected dose were investigated (Shiri et al. 2020b). This includes reduction of acquisition time per projection (or low-dose imaging) and reduction of the number of angular projection (fast SPECT imaging). A deep learning algorithm was then employed to predict the full time and missing angular projections from the SPECT data in the projection space. The deep learning solution was able to effectively retrieve the standard image quality at the cost of slight quantification bias.

## Radiation dosimetry calculations

The applications of AI in internal radiation dosimetry and molecular radiotherapy deal mostly with organ and tumor delineation/segmentation, tissue and lesion characterization, absorbed dose estimation, and therapeutic dose calculation. Lesion and organ delineation/segmentation described in the previous section is equally employed in dosimetry and radiation therapy to calculate clinically relevant parameters as well as targeting specific organs/lesions to deliver the prescribed therapeutic doses (Sahiner et al. 2019). Tissue and lesion characterization, mostly employed for computer-aided diagnosis and outcome prediction, will be addressed in the following section.

In radiation oncology, AI approaches could assist in treatment planning, adaptation, and assessment of response to therapy (Sahiner et al. 2019). The accurate estimation of the dose distribution enables effective clinical plan optimization to save time and maintain high-quality treatment plans. To this end, deep learning approaches were employed to predict the absorbed dose within the contours of the planning target volume (PTV) and organs at risk (OARs) for prostate cancer patients (Nguyen et al. 2019). A multi-channel deep neural network is fed with the CT image along with the contours of the PTV and OARs separately, which resulted in accurate prediction of the dose distribution from intensity-modulated radiation therapy. Moreover, historical treatment plans (prior information) have been incorporated into a deep reinforcement learning framework to develop an automated protocol adaptation in radiation therapy of non-small cell lung cancer (Tseng et al. 2017). The aim was to maintain maximum tumor local control while reducing the rate of radiation pneumonitis grade. AI-based solutions are also being exploited to estimate the toxicity imposed to normal tissues and organs in order to better understand the dose-toxicity relationship for safe dose escalation (Zhen et al. 2017).

AI approaches could assist with patient positioning and tracking of internal tumor/organ motions in real-time plan adaptation. In this context, deep learning approaches enabled dynamic tracking of lung tumors to specify anatomical position/shape of the target from a single radiographic projection in real-time (Foote et al. 2018; Zhao et al. 2019b) or estimate patient-specific volumetric CT images from a single projection data (Shen et al. 2019).

As discussed in the previous section, AI approaches are used to achieve cross-modality image translation, in particular MRI to CT image synthesis, to take advantage of MRI in both clinical diagnostic and therapy planning. This approach is useful in MRI-only or PET/MRI-guided radiotherapy for real-time adaptive re-planning, wherein the rapid generation of synthetic CT images is highly demanded for dose calculation (Sahiner et al. 2019; Maspero et al. 2018; Largent et al. 2019).

**Fig. 5:**
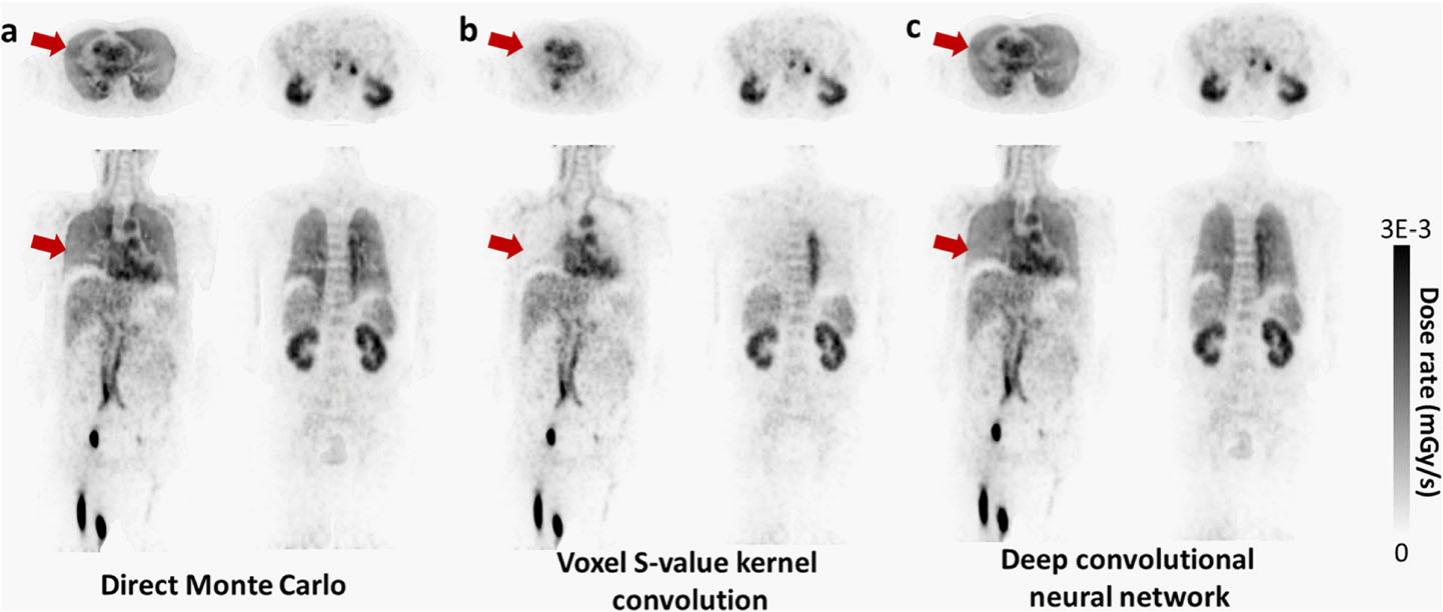
Dose rate maps estimated by (**a**) direct Monte Carlo, (**b**) VSV kernel convolution, and (**c**) deep convolutional neural network. Reprinted with permission from Springer Nature (Lee et al. 2019)

Owing to the remarkable growth in personalized medicine, highly accurate patient-specific dosimetry calculations are crucial in molecular radiotherapy. State-of-the-art Monte Carlo (Liu et al. 2017) simulation for voxel-based dosimetry is considered as the most reliable technique in personalized dosimetry. Nonetheless, MC simulation requires prohibitively long computational time and resources. As such, it is seldom utilized in clinical practice. Deep learning approaches have been investigated to cope with this challenge through introducing a neural network to replace MC-based dose estimation. Voxel-wise absorbed dose prediction from PET imaging was achieved via training a deep neural network, wherein PET and CT images were fed into a deep learning network as input to predict the corresponding dose rate map (Lee et al. 2019). For this purpose, direct MC simulation was considered as ground truth. The deep learning-based dose prediction model exhibited superior performance over conventional dosimetry methods demonstrating good agreement with results achieved through direct MC simulations. Figure 5 shows an example of direct Monte Carlo-based dose estimation from PET imaging compared to voxel *S* value (VSV) kernel convolution and deep learning-based approaches. Good agreement between Monte Carlo simulation and deep learning results can be observed, particularly in the lung region.

## Computer-aided diagnosis and outcome prediction

Machine learning techniques have been exploited over the past decade to aid clinical diagnosis and characterization of diseases through a process known as computer-aided diagnosis. In this regard, radiomic features have been widely used for prognosis, cancer subtyping, and lesion/tissue characterization (Visvikis et al. 2019). Radiomic features extraction can be carried out in a hand-crafted or engineered manner, though recently deep learning-based (automated) feature extraction has shown promise through superior discriminative and indicative feature selection (Sollini et al. 2019). Hand-crafted radiomic features devised depending on the characteristics of the imaging modality to aid the interpretation of medical images are commonly fed into a classifier to predict the risk of cancer development, aggressiveness of tumors, and likelihood of malignancy (Giger et al. 2013). Conversely, deep learning-based approaches take a sub-region of an image as input followed by feature extraction optimization (with minimal human intervention) to maximize the accuracy of outcome prediction (González et al. 2018).

Lesion characterization using AI algorithms is mainly conducted to describe a lesion behavior over time, for instance, to specify false-positive diagnosis. In addition, lesion characterization is also used in imaging genomics (concerned with the function, structure, and evolution of genomes), wherein radiomic features extracted from the lesion are regarded as phenotypes to investigate the correlation and/or association with histopathology. Deep learning-based lesion analysis (serving as feature extractor) is not intuitive, as opposed to handcrafted features, wherein features associated with certain known traits are specified/established during a supervised training scheme. These features, extracted/selected via a supervised learning process could subsequently be used for further genomic discovery studies (Burnside et al. 2016; Sun et al. 2017).

In addition to malignant lesions, tissue characterization is sought when the focus is on non-malignant tissue to assess the possibility of disease development, for instance, parenchyma analysis with the goal to assess the risk of breast cancer (Li et al. 2012). In this regard, deep learning approaches have been exploited to relate the breast density and parenchymal patterns to the risk of breast cancer development (Lee and Nishikawa 2018). Similarly, patches of lung tissue have been used to train deep learning algorithms to categorize interstitial lung disease into normal tissue, consolidation, micro-nodules, reticulation, … etc (Sim et al. 2020; Anthimopoulos et al. 2016).

In myocardial perfusion SPECT imaging, Betancur et al. proposed a deep learning-based automated prediction/detection of obstructive disease and compared its performance to commonly used total perfusion deficit (TPD) index (Betancur et al. 2018). A large cohort of patients (> 1600) in a multicenter study setting was processed by a deep learning method to predict coronary artery disease per patient and per vessel. Overall, the results suggested that deep learning prediction outperformed conventional TPD approach.

**Fig. 6:**
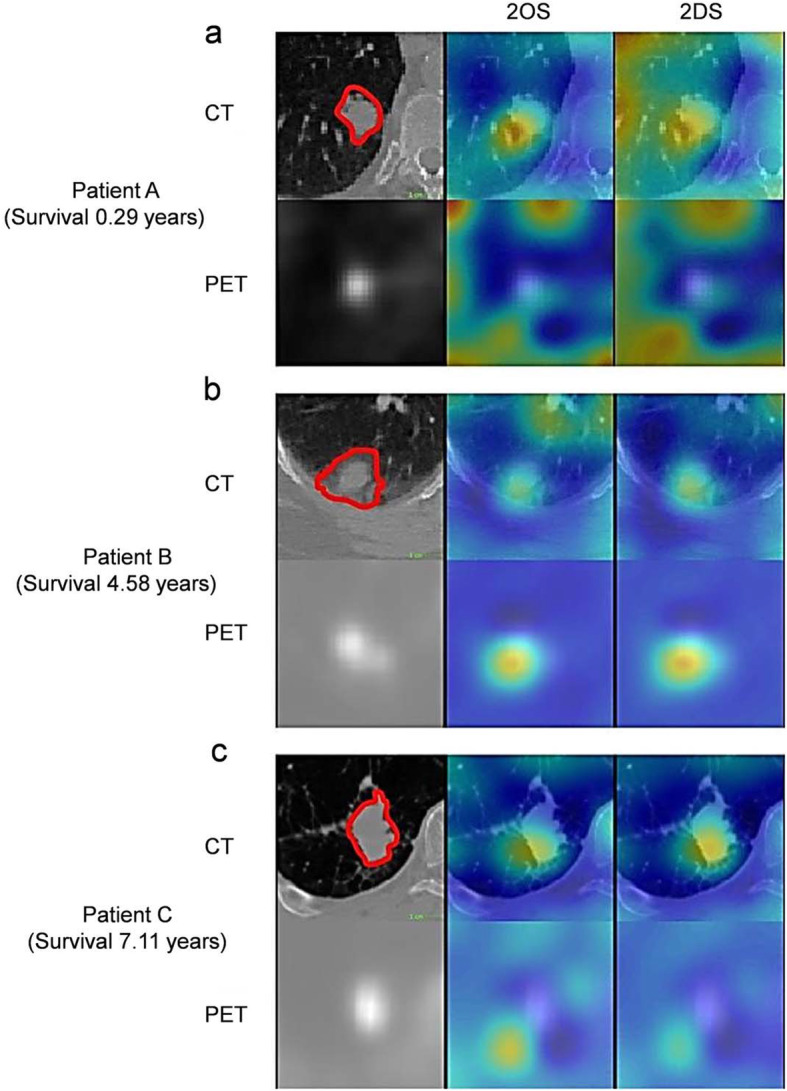
Visualization of the U-Net features. Regions that predicted death of the patients obtained via a guided back-propagation method (Yosinski et al. 2015). Trivially, tumoral regions are highlighted in red in the heatmap. However, some of the heated regions outside of the tumoral volume matched with the actual locations of recurrences and metastases when they were compared with the post-therapeutic images and clinical records, rendering a great potential as a practical, clinical tool for patient-tailored treatment planning in the future. **a** Patient deceased in 0.29 years after the acquisition of the images. **b** Deceased after 4.58 years. **c** Deceased after 7.11 years. 2OS and 2DS stand for 2-year overall survival and 2-year disease-specific survival, respectively. Reprinted with permission from Springer Nature (Baek et al. 2019)

Computer-aided diagnosis frameworks aim at characterizing tissues or lesions to estimate the probability of malignancy (which can be considered as a classification task). Breast cancer has been an active research topic for the development of AI-based solutions to discriminate between malignant and benign lesions (Zheng et al. 2020). In this regard, combining conventional radiomics-based computer-aided diagnosis with deep learning-based feature fusion resulted in statistically significant enhanced level of diagnostic accuracy (Antropova et al. 2017). Deep learning methods are increasingly exploited to provide additional decision support in the diagnosis of different disease types, such as lung cancer (Gao et al. 2018; Lessmann et al. 2018; Sibille et al. 2020) where conventional radiomics-based approaches are providing promising results. Baek et al. claimed that deep learning algorithms are capable of providing an enhanced predictive power compared to hand-crafted radiomic features (Baek et al. 2019). They trained a U-net model for tumor segmentation relying only on manually defined contours as ground truth. They discovered a rich set of highly discriminative image features related to the patients’ survival with exceptional prognostic value. Figure 6 depicts the voxel-wise head-maps of back-propagated gradient values wherein the high correlation is observed between probability of death and gradient values.

## Challenges and opportunities

Most AI-based methodologies proposed for use in clinical practice deal with some sort of automation in terms of quantification, segmentation, detection, and diagnosis. In this regard, the major concern is who is responsible/accountable or what would happen if AI-based systems misdiagnose or fail? To address this issue, we should first answer this question, do radiologists/physicians and other medical experts ever make mistakes? If yes, then, similar strategies could be adopted to tackle the issue of failure in AI-based frameworks. In this light, there is a consensus that in short runs combination of AI-based frameworks with complementary human intervention could result in synergistic effects in patient management, interpretation, and diagnosis.

Moreover, a distinction should be drawn between replacement of conventional or current algorithms/systems/frameworks with AI-based solutions and replacement of experts/clinicians/human resources with AI-based solutions. At present, some AI-based approaches have exhibited adequate accuracy and robust performance to be employed as an alternative or complementary resource to conventional tools in clinical practice (Hsieh et al. 2019). In this regard, failure of AI-based approaches can be verified/corrected by existing conventional tools, though human intervention would be required. Conversely, replacement of experts and human interpretation with current AI-based solutions is still far from full-scale implementation in clinical setting. Currently, AI-based solutions could only assist experts to create a synergy between humans’ expertise and machines’ capacity. Incidentally, potential failure of AI-based solutions can be corrected/ignored by the experts.

**Fig. 7:**
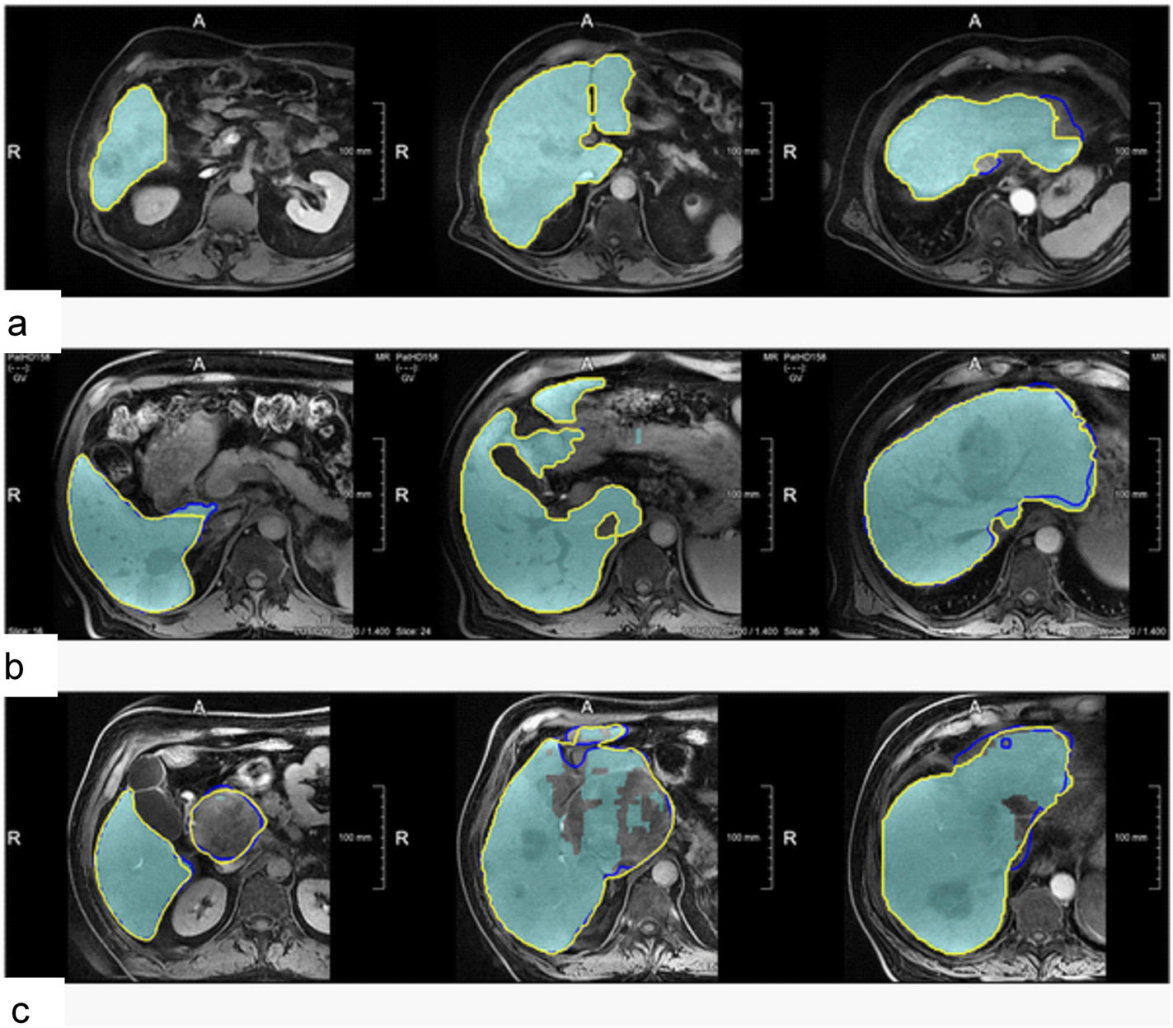
Examples showing how the automatic Liver-Net results (cyan) were corrected. The contours denote corrected liver masks by radiology residents with 3-year experience (Res3) (blue) and radiology assistant (RA) (yellow). **a** Case with minor or no corrections: 1.42% for the Liver-Net liver masks, 1.51% (4.3%), and 1.42% (0%) mean relative error (RVE) percentage of corrected slices (CS) for the corrections by Res3 and RA, respectively. **b** Case where less than half of all slices were corrected: 5.66%, 5.57% (47.6%), and 5.40% (35.7%). **c** Case where most of the slices were corrected by all observers: 11.48%, 1.62% (74.0%), and 0.10% (78.3%). Reprinted with permission from the Public Library of Science (Chlebus et al. 2019)

In case of failure of AI-based solutions, there is no straightforward framework to fix the outcome for a specific clinical study owing to the black-box nature of AI algorithms. Hence, there should be alternative tools to verify/correct the outcome of AI-based techniques. In the long term, adaptive and/or interactive training schemes should be devised to improve the performance of AI algorithms considering the users’ feedback or through regular training updates using larger datasets to enhance the robustness of AI-based solutions or reduce the likelihood of failure (Chlebus et al. 2019; Boers et al. 2020; Tang et al. 2020). Chlebus et al. compared manual corrections of liver delineation on MR images performed by a deep learning network (Liver-Net) with manual routine segmentations in terms of inter-observer variability (Chlebus et al. 2019). Figure 7 illustrates representative examples of manual correction of the automated produced contours by the Liver-Net wherein the resulting liver masks exhibited a significantly lower intra-observer variability compared to manual routine liver delineation with mean relative errors of 0.69% and 2.75%, respectively.

Despite promising results repeatedly reported regarding the application of AI-based methodologies, these approaches are still moving from “proof of concept” phase into more practical applications. The majority of AI-related works in the literature report on single-institution efforts under controlled conditions (e.g., diversity of patient population or image quality). The challenge of performance/bias assessment of AI approaches under realistically diverse conditions (e.g., multi-center studies) warrants further investigation. The performance of AI algorithms depends largely on the training data used for model development. As such, the analysis of risks associated with the deployment of AI-based methods when exposed to a different test dataset to ensure that the developed model has sufficient generalizability is an important part of quality control measures that need to be implemented prior to their use in the clinic.

Data collection, a critical step in AI solutions, is one of the major challenges faced by developers. Though there are uncountable numbers of clinical databases around the globe, many of them are not valid or properly annotated as required by learning systems. To address this challenge, labor-intensive efforts have to be made by specialists/researchers to create dependable datasets large (in terms of number of subjects) and diverse (covering a realistic and representative range of clinical cases) enough for AI solutions. Hence, in addition to efforts made to create robust AI models, large scale collaborations are required to create and maintain such databases. For some applications, such as denoising or image reconstruction, collection of training data through simulation or experimental phantom studies may be less challenging. However, simulation studies might not fully represent practical scenarios wherein the noise, intra- and inter-patient variability, complex physical factors, and unpredictable errors, such as patient motion or presence of abnormalities or anomalies would challenge AI algorithms in clinical setting. Extensive phantom studies and Monte Carlo simulations are required to develop robust and versatile AI-based solutions for these applications.

AI approaches and in particular deep learning methods have witnessed impressive progress over the past few years showing great promise for future applications in molecular imaging. Though AI methods still have a long way to go to play a major role in clinical practice and undertake part of the radiologists’ responsibilities (Hustinx 2019; Mazurowski 2019; Yi et al. 2018), it is time to define and introduce the frameworks, protocols, and standards to exploit these approaches as an alternative option or to assist processes and decisions taken in clinical practice. The performance of AI approaches could equal or even surpass human/specialist’s performance in a variety of applications in medicine. Nevertheless, most of the challenges in medical imaging, diagnosis, and therapy are still far from being completely solved, which necessitates further task-based development/optimization of AI algorithms/architectures. To this end, each aspect of the AI triangle should be adequately established. This triangle consists of big data (covering wide and realistic range of subjects), algorithm/architecture, and processing power. Though a number of efforts set out to alleviate the issue of limited data size, big data is still deemed the major challenge of AI to draw a comprehensive picture of its potential and pitfalls in medical practice. Moreover, the notion of big data is a vague concept, which remains to be outlined empirically owing to its great task dependency.

## Data Availability

Yes (own data and material)
